# Using a Feedback Environment to Improve Creative Performance: A Dynamic Affect Perspective

**DOI:** 10.3389/fpsyg.2017.01398

**Published:** 2017-08-15

**Authors:** Zhenxing Gong, Na Zhang

**Affiliations:** ^1^School of Business, Liaocheng University Liaocheng, China; ^2^Beijing Information Science and Technology University Beijing, China

**Keywords:** feedback environment, dynamic affect, creative performance, positive affect, negative affect

## Abstract

Prior research on feedback and creative performance has neglected the dynamic nature of affect and has focused only on the influence of positive affect. We argue that creative performance is the result of a dynamic process in which a person experiences a phase of negative affect and subsequently enters a state of high positive affect that is influenced by the feedback environment. Hierarchical regression was used to analyze a sample of 264 employees from seven industry firms. The results indicate that employees’ perceptions of a supportive supervisor feedback environment indirectly influence their level of creative performance through positive affect (t2); the negative affect (t1) moderates the relationship between positive affect (t2) and creative performance (t2), rendering the relationship more positive if negative affect (t1) is high. The change in positive affect mediates the relationship between the supervisor feedback environment and creative performance; a decrease in negative affect moderates the relationship between increased positive affect and creative performance, rendering the relationship more positive if the decrease in negative affect is large. The implications for improving the creative performances of employees are further discussed.

## Introduction

The creative performance of employees is the primary means for promoting innovation, enhancing competitiveness, and developing an enterprise’s competitive advantage (Zhou and Shalley, 2008). Because environmental factors are more likely to promote intervention and the effect of environments can be perceived within a short period, environmental factors have become the current research hotspot in the field of creativity performance. Among the mechanisms of environmental influence on creative performance, affective states are prominent as factors that can be influenced and that have consistently been identified as links between environment and creative performance (Baas et al., 2008). Prior research has confirmed that feedback is an important environmental factor (Ford, 1996; Zhou and George, 2001) that can influence creative performance through the mediating role of affect; however, the results are inconsistent. For example, some studies have shown that feedback can positively influence creative performance via the mediating role of positive affect, which encompasses feelings such as happiness and enthusiasm (Madjar et al., 2002). Other studies have found that feedback can positively influence creative performance through the mediating role of negative affect (Baumann and Kuhl, 2002). This inconsistency in research results may suggest that managers are unaware of the need to give employees feedback to influence positive or negative affect and thus improve creative performance; thus, resolving the inconsistent results regarding the affect mechanism between feedback and creative performance has become a key research focus.

Researchers have identified several gaps in the results regarding the affect mechanism between feedback and creative performance. First, it is questionable whether feedback intervention can reflect the entire relationship between feedback and affect. Researchers should consider the broader psychological feedback context in which these feedback interventions occur as a single complex process (Ashford and Northcraft, 2003; Levy and Williams, 2004). It is difficult to fully understand how feedback affects work-related outcomes through affect when focusing on only isolated feedback interventions. Second, it is questionable whether static affect can reflect the actual influence mechanism. A person’s affect continuously changes in response to feedback, and changes in positive and negative affect have critical effects on creative performance (Judge and Ilies, 2004). If this is the case, a study focusing on only static affect may fail to explain the mechanism. Third, it is also questionable whether positive affect alone promotes creative performance. As one of the most complex mental functions, creative performance may draw from the entire spectrum of affective experiences (George and Zhou, 2007), thus a one-sided focus on positive affect may not fully explain creative performance.

For solving these limitations, our study aims to make several contributions to the literature on the feedback–creative performance relationship. First, we use feedback environment to instead feedback intervention. To accurately reveal the relationships among feedback, affect and creative performance, it is necessary to examine feedback as a multidimensional structure from the perspective of integration. In place of feedback, we consider the feedback environment, which refers to the contextual processes between the supervisor and subordinate or between coworker and coworker in the daily work environment rather than organizing a formal performance appraisal feedback session (Steelman et al., 2004). Compared with feedback, feedback environment can lead more consistent expected results, such as performance, identification, and organizational citizenship behavior (Anseel and Lievens, 2007). In the case of supervisors and coworkers, the supervisors play a greater role in influencing creative performance (George and Zhou, 2007). In the current study, we place the supervisor feedback environment in the fundamental position, similar to previous studies (Levy and Williams, 2004; Anseel and Lievens, 2007).

Second, we assess the role of different affect changes in the relationship between feedback environment and creative performance, and explore the affect mechanism from the perspective of dynamic affect. Although theories of self-regulation emphasize that change in affect and the interplay of positive and negative affect have critical functions (Kuhl, 2000), previous research has ignored this theoretical notion (Madjar et al., 2002). Affect changes that could compensate for static affect research reflect the particular form of emotional change and dynamic ambivalence and present the true state of individual work in daily life (Bledow et al., 2013). Different affect changes can have different effects on creative performance. If negative affect decreases, the focus of cognitive processing expands, which enables associations among remotely connected concepts (Baumann and Kuhl, 2002). Because of an increase in positive affect after an episode of negative affect, a person reaches a state of high positive affect that enables a flexible mode of thinking that can lead to creative performance.

Third, we emphasize the role of negative affect and argue that creative performance is a consequence of a dynamic process in which one experiences a phase of negative affect and subsequently leaves it behind and enters a highly positive state (Baas et al., 2008). Affect is one of the most complex mental functions, so a one-sided focus on positive affect cannot fully explain the mechanism about creative performance (George and Zhou, 2007). However, neglecting this theoretical notion, empirical research has mostly considered isolated affective states rather than the dynamic interplay of positive and negative affect as a determinant of creative performance.

Thus, the primary purpose of this research is to resolve these problems by analyzing how the feedback environment affects creative performance through positive affect (t2) and positive affect changes and to analyze the moderating role of negative affect (t1) and negative affect changes.

## Theory and Hypotheses

### Feedback Environment and Creative Performance

Steelman et al. (2004) deemed that the feedback environment comprises seven facets: source credibility, feedback quality, feedback delivery, favorable feedback and unfavorable feedback that accurately reflect performance, source availability, and support for seeking feedback. Source credibility is conceptualized as the expertise and trustworthiness of the feedback source. Consistency and usefulness have been demonstrated to be important aspects of feedback quality. A feedback recipient’s perceptions of the source’s intentions in giving feedback will affect his or her reactions and responses to the feedback. Favorable or unfavorable feedback is conceptualized as the perceived frequency of positive/negative feedback when, from the feedback recipient’s perspective, his or her performance does in fact warrant positive or negative feedback. Supervisor source availability is operationalized as the perceived amount of contact an employee has with his or her supervisor and the ease with which feedback can be obtained. Feedback-seeking promotion is defined as the extent to which the environment is supportive or unsupportive of seeking feedback (George and Zhou, 2007).

First, the primary literature has shown that some dimensions of the feedback environment influence the employees’ creative performance. For instance, with regard to feedback delivery, research has indicated that if feedback is delivered supportively, then the purpose for informing employees about their contribution is more apparent; thus, the likelihood of improving creative performance is increased (Zhou, 1998). Second, an upstanding feedback environment is both supportive and incentivizing, which improves creative performance. The combination of these facets is believed to reflect a balanced feedback environment. These seven dimensions compose a highly supportive feedback environment (Whitaker et al., 2007), which may make the employees feel appreciated and cultivate support for leadership. Such support may motivate employees to approach their work in a more positive manner and increase the salience of feedback and the importance of the feedback process (Sparr and Sonnentag, 2008). In laboratory and field research, a positive and stimulating work environment is connected with creativity, and a non-supportive work environment is negatively associated with creativity (Shalley and Zhou, 2008). Recipients of feedback recognize how information helps them take control of their own creative performance (Shin and Zhou, 2003; Amabile et al., 2004). Considering the above arguments, we offer the following assumptions:

Hypothesis 1: A supportive supervisor feedback environment (t1) relates positively to creative performance (t2).

### Feedback Environment, Affect, and Creative Performance

An organization is full of affect, and complicated affect determines what employees like to do. Work processes and results make employees feel happy, anxious, or depressed. Studies have determined that creative performance is shaped by affect experience and forms complicated affect experiences (Amabile, 1996). Work events lead to different experiences of affect and then influence changes in individual behavior; thus, affect experiences play the core role in how work events affect individual behavior (Weiss and Cropanzano, 1996).

Research indicates that people who receive positive feedback are more satisfied and experience positive affect because they believe the feedback content is accurate; however, people who receive negative feedback and criticism suspect that the feedback is not accurate and have negative affect reactions (Ilgen et al., 1979). Feedback environment is a comprehensive concept comprising both positive and negative feedback and emphasizing feedback accuracy. Even people who receive negative feedback have negative affect reactions, when he/she feels the accuracy about negative feedback, they would be more satisfied and experience positive affect. Thus, the feedback environment includes not only the function of valence but also the accuracy of the judgment (Steelman et al., 2004). Hence, we propose the following hypotheses:

Hypothesis 2: A supportive supervisor feedback environment (t1) relates positively to positive affect (t2).
Hypothesis 3: A supportive supervisor feedback environment (t1) relates negatively to negative affect (t2).

The dynamics of affect enable the integration of cognitive functions that are necessary for creative performance (Bledow et al., 2013). Affect is one of many sources, and affect change is a mixture of affect, observation and the recognition of contradiction that changes the individuals focus, thinking mode, and creative performance (Amabile et al., 2005). Prior research has shown that positive affect increases the likelihood that new and useful ideas will be developed (Baas et al., 2008). Positive affect leads to creative performance because it activates cognition and increases cognitive flexibility (De Dreu et al., 2008).

Negative affect can lay the foundation for creative performance. De Dreu et al. (2008) observed that the experience of negative affect generated new ideas because it prompted participants to show greater persistence at the task. If the level of positive affect is high and the context is supportive, the level of negative affect is positively related to creative performance (George and Zhou, 2007). However, negative affect contributes to creative performance through a delayed process that depends on subsequent positive affect (Bledow et al., 2013). Negative affect focuses on problems that require effort to resolve (Foo et al., 2009). An episode of negative affect is associated with a bottom-up mode of cognitive processing that focuses on inconsistent and unexpected information (Kuhl, 2000) and through which an objective understanding of a situation can be developed (Spering et al., 2005). During a subsequent episode of positive affect, cognitive flexibility and activation increase, and knowledge is processed in a top-down manner (Baumann and Kuhl, 2002). Positive affect enables creativity, and new ideas likely emerge during an episode of positive affect. However, without negative affect to lay the foundation for new ideas, positive affect is likely to be weaker. Hence, we propose the following:

Hypothesis 4: Positive affect (t2) mediates the relationship between supervisor feedback environment (t1) and creative performance (t2).
Hypothesis 5: Negative affect (t1) moderates the relation between positive affect (t2) and creative performance (t2) such that the relation is more positive if negative affect (t1) is high.

### Feedback Environment, Affect Changes, and Creative Performance

Changes in the environment affect an individual’s perception of uncertainty, and that perception provides the raw material for subsequent meaning construction processes. That is to say, changes in the environment affect the variation, which is the result of the interaction between the individual and the environment. Variation is the result of previous experience. Affect changes comprise information processing and changes in individual cognition, which leads to the individual exhibiting different behaviors (Ford and Kuenzi, 2008). By providing feedback information that is reflects an accurate assessment of an individual’s creative performance and by having effective standards, the feedback environment can induce instant positive and negative affect. Subsequently, employees may determine the accuracy of negative feedback; thus, negative affect may be shifted, and positive affect can increase. As the result of an increase in positive affect after an episode of negative affect (Solomon and Corbit, 1974), a person reaches a state of high positive affect that enables a flexible mode of thinking that can lead to creative performance (Bledow et al., 2013). Hence, we propose the following hypotheses:

Hypothesis 6: A supportive supervisor feedback environment (t1) relates positively to an increase in positive affect (t2).
Hypothesis 7: A supportive supervisor feedback environment (t1) relates positively to a decrease in negative affect (t2).

Affect changes involve an increase in positive affect and a decrease in negative affect from t1 to t2. An increase in positive affect from t1 to t2 is an important component of affective change because positive affect at t1 will not be at a high level because of the presence of negative affect (Schmukle et al., 2002). As a result of an increase in positive affect after an episode of negative affect, a person reaches a state of high positive affect that enables the flexible mode of thinking that can lead to creative performance. The creative performance-enhancing effect of positive affect unfolds over time and is more pronounced during longer episodes of positive affect than during shorter ones (Amabile and Staw, 2005). An increase in positive affect should be more strongly related to creative performance if that increase is accompanied by a decrease in negative affect. Although negative affect can lay the foundation for creativity at a later point in time, at any given moment, the presence of negative affect impedes rather than enables creative performance (Baumann and Kuhl, 2002). Negative affect inhibits remote associations, which are an important component of creative performance. If negative affect decreases, the focus of cognitive processing expands, which enables associations among remotely connected concepts (Baumann and Kuhl, 2002). The activation of a person’s associative networks of memory is strongest after a decrease in negative affect; that is, activation is stronger if negative affect is first experienced and then down-regulated, as opposed to a situation in which no negative affect was present (Kuhl, 2000). Because of the activation of associative networks of memory, a decrease in negative affect should broadly facilitate new associations so that the individual can develop new ideas that are not constrained by the cognitive content that he or she focused on before the decrease in negative affect. High creativity should thus result if an increase in positive affect is accompanied by a decrease in negative affect. An increase in positive affect leads to higher cognitive activation and flexible top-down processing of existing knowledge. A decrease in negative affect activates associative networks of memory and enables the integration of information that was processed in a bottom-up manner during an episode of negative affect (Baumann and Kuhl, 2002).

Hypothesis 8: An increase in positive affect mediates the relationship between the supervisor feedback environment (t1) and creative performance (t2).
Hypothesis 9: A decrease in negative affect moderates the relationship between an increase in positive affect and creative performance, and the relationship is more positive if the decrease in negative affect is high.

**FIGURE 1:**
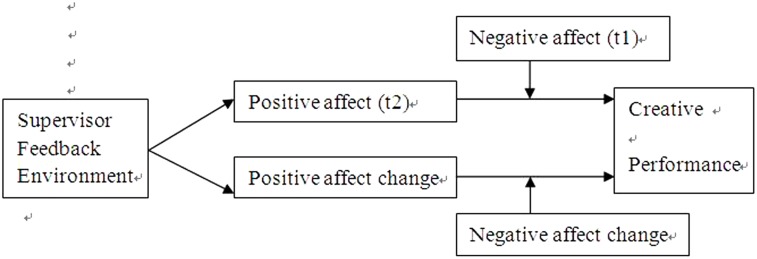
Test model.

We have developed moderated mediation hypotheses and augmented our research model (**Figure 1**).

## Materials and Methods

### Participants and Procedures

This study is based on the principle of combining random sampling and convenience sampling. Valid samples were screened according to the following assumptions: the sample participants were volunteers, work requires new ideas, two surveys can match, and the feedback environment influences affect. The valid sample comprised 264 employees from seven industries that were not multinational corporations. Demographic information indicated that there were 134 male employees (50.8%) and 130 female employees (49.2%) in the sample. More than 89% of the employees were 20–35 years of age. Of the participants, 68.2% held a bachelor’s degree or above, 73.8% had worked for fewer than 3 years, and 92.8% had worked for fewer than 5 years.

Data collection was divided into two components. First, the participants filled out a questionnaire to measure demographic control variables, the supervisor feedback environment, and positive and negative affect upon arriving at their office and before starting work. Second, the participants filled out a questionnaire to measure PANAS (Positive Affect and Negative Affect Scale) and creative performance before the scheduled end of their work on the same day.

### Ethics Statement

This study was reviewed and approved by of American Psychological Association Ethics Committee Rules and Procedures, APA Ethics Committee with written informed consent from all participants. All participants gave written informed consent in accordance with the Declaration of Helsinki.

### Instruments

The measurement items used in the present study were primarily developed in English; to ensure cross-linguistic equivalence, we translated all scale items into Chinese and then translated them back into English using two bilingual (English–Chinese) professional translators (Brislin, 1980).

#### Supervisor Feedback Environment

We measured the supervisor feedback environment using Steelman et al.’s (2004) scale. This Likert scale assesses each feedback environment dimension and the seven facets within each dimension. An item from the Supervisor Feedback Environment scale is “My supervisor generally lets me know when I do a good job at work.” The combination of these facets is believed to reflect a balanced feedback environment. These seven dimensions compose a highly supportive feedback environment, which may make the employees feel appreciated and cultivate support for leadership (Whitaker et al., 2007). Because the hypotheses in this study operate at the construct level, like prior research (Steelman et al., 2004), our analyses used a composite score of the feedback environment rather than a score based on the individual facets. The Cronbach’s α for the measure of the supervisor feedback environment was 0.93.

#### Positive and Negative Affect

Positive and negative affect were measured as psychological states using the PANAS inventory each morning and at the end of each work day (Watson et al., 1988). In the morning survey, the instructions were “Please indicate how you feel this morning,” and the participants were asked to report their affective state regarding each adjective on a 5-point scale. The Cronbach’s alphas were 0.87 for the positive affect scale and 0.88 for the negative affect scale. At the end of the work day, the participants reported their affective state in relation to identical positive and negative affect adjectives (Cronbach’s alphas of 0.76 and 0.78, respectively). The participants were instructed to indicate how they felt, on average, during that work day. To calculate the change in affect, we used residual change methods such as those reported by Förster and Higgins (2005). Residual change refers to the deviation of actual t2 values from those that would be expected based on t1 values.

#### Creative Performance

To avoid common method bias, in a separate questionnaire, each employee’s supervisor rated the employee’s creativity like prior research (Zhou and George, 2001; Zhang et al., 2017). The supervisors assigned to complete the rating forms were those who had plenty of opportunity to observe their employees’ creative performance. Consistent with prior studies, we used supervisor ratings to assess the creative performances of employees (Zhou and George, 2001). Using 13 items, each supervisor rated how often their subordinates displayed creative behavior in the workplace on a 5-point scale ranging from never to always. A sample item from the scale reads “Seeks out new technologies, processes, techniques and/or product ideas.” The Cronbach’s α for the measure of creative performance was 0.92.

#### Controls

Consistent with previous creativity research (Zhou and George, 2001), we controlled for demographic variables that have been determined to be significantly related to creativity: age, gender, job tenure, and education.

## Results

**Table 1** presents the means, standard deviations, reliability coefficients, and correlations among the study variables. An inspection of the correlations reveals that supervisor feedback environment is positively related to positive affect (t2; *r* = 0.27, *p* < 0.01), positive affect change (*r* = 0.17, *p* < 0.01) and creative performance (*r* = 0.68, *p* < 0.01). The results also indicate that positive affect (t2; *r* = 0.46, *p* < 0.01), positive affect change (*r* = 0.22, *p* < 0.01), negative affect (t1; *r* = 0.50, *p* < 0.01), and negative affect change (*r* = -0.42, *p* < 0.01) are significantly related to creative performance.

**Table 1:** Means, standard deviations, and correlations of all measures.

	Mean	SD	1	2	3	4	5	6	7	8
1. Supervisor feedback environment	2.36	0.72	–							
2. Positive affect (t1)	2.21	0.66	0.18*	–						
3. Negative affect (t1)	2.14	0.60	0.17*	-0.79**	–					
4. Positive affect (t2)	2.09	0.69	0.27**	0.79**	-0.74**	–				
5. Negative affect (t2)	1.94	0.58	-0.09	-0.76**	0.71**	0.80**	–			
6. Positive affect change	0.30	0.42	0.17*	0.26**	0.20**	0.62**	0.31**	–		
7. Negative affect change	0.59	0.18	-0.10	-0.17	-0.21**	-0.5**	0.79**	-0.17*	–	
8. Creative performance	2.71	1.12	0.68**	0.41**	0.50**	0.46**	0.42**	0.22**	-0.42**	–
9. Gender	–	–	-0.37**	-0.09	-0.11	-0.1	-0.08	-0.04	-0.09	-0.1
10. Age	1.66	0.67	-0.07	-0.03	-0.09	-0.14	-0.06	-0.18	-0.06	0.02
11. Job tenure	2.16	0.79	-0.21**	0.03	0.00	0.04	0.05	0.03	0.05	0.09
12. Education	–	–	0.13	-0.03	-0.05	0.15	0.03	0.06	0.03	0.26


*n = 264; ^∗^p < 0.05, ^∗∗^p < 0.01*.

Further analyses were conducted to better estimate the overall contribution of the supervisor feedback environment to creative performance and the mediating role of positive affect (t2)/positive affect change. We adopted the procedure proposed by Preacher and Hayes (2008). According to their suggestions, there are three criteria that justify a mediation effect. First, the independent variable should be significantly correlated with the mediator variable. Second, after the effect of the independent variable on the dependent variable is controlled, the correlation between the mediator variable and the dependent variable should be significant. Finally, the indirect effect of the independent variable on the dependent variable must be significant. Before the analyses, all continuous predictors were well centered (Aiken et al., 1991).

**Table 2** summarizes the hierarchical regression results. After controlling for the effects of participant demographics, the supervisor feedback environment significantly predicted positive affect (t2; model 2: β = 0.17, *p* < 0.01), positive affect change (model 4: β = 0.12, *p* < 0.01) and creative performance (model 1: β = 0.59, *p* < 0.01) but did not significantly predict negative affect (t2) (model 3: β = -0.07, n.s.) or negative affect change (model 5: β = -0.13, n.s.). Taken together, Hypotheses 1, 2, and 6 received support. Hypotheses 3 and 7 were not supported.

**Table 2:** Hierarchical regressions for the impact of supervisor feedback environment on affect, affect change, and creative performance.

Independent variable	Dependent variable				
	Model 1	Model 2	Model 3	Model 4	Model 5
	Creative performance	Positive affect (t2)	Negative affect (t2)	Positive affect change	Negative affect change
Supervisor feedback environment	0.59**	0.17**	-0.07	0.12**	-0.13
Gender	0.06	-0.15	-0.13	-0.05	-0.04
Age	-0.21	-0.45**	-0.24**	-0.32**	-0.07**
Job tenure	0.25	0.36**	0.20**	0.25**	0.06**
Education	-0.66	0.06	0.06	0.06	0.02
*R*^2^	0.08	0.12	0.04	0.15	0.05
Δ*R*^2^	0.07	0.1	0.03	0.13	0.03
*F*	4.97**	7.06**	2.63*	8.73**	2.63*


*n = 264; ^∗^p < 0.05, ^∗∗^p < 0.01*.

As shown in **Table 3**, after positive affect (t2) was taken into account, the effect of the supervisor feedback environment on creative performance (model 1: β = 0.05, n.s.) became non-significant; however, the effect of positive affect (t2) on creative performance (model 1: β = 0.76, *p* < 0.01) remained significant. After the positive affect change was considered, the effect of the supervisor feedback environment on creative performance (model 3: β = 0.06, n.s.) became non-significant; however, the effect of positive affect change on creative performance (model 3: β = 0.56, *p* < 0.01) remained significant. To calculate the indirect effects, we adopted the SPSS micro PROCESS (Hayes, 2013). The results presented in **Table 4** indicate that the formal two-tailed significance test (assuming a normal distribution) show that the indirect effect was significant. Bootstrapping results confirmed the Sobel test, with bootstrap 95% confidence intervals of 0.04–0.23 and 0.02–0.13 around the indirect effect not containing 0. Considered together, Hypotheses 4 and 8 received full support.

**Table 3:** Hierarchical regressions for testing mediator and moderator.

	Model 1	Model 2	Model 3	Model 4
Intercept	1.15**	2.63**	2.41**	2.51**
Supervisor feedback environment	0.05	0.03	0.06	0.09
Positive affect (t2)	0.76**	0.29*	0.56**	
Negative affect (t1)		0.71**		
Positive affect × negative affect (t1)		0.37*		
Positive affect change				0.28*
Negative affect change				-0.32*
Positive affect change × negative affect change				-0.72**
Δ*R*^2^	0.18	0.07	0.02	0.05
*F*	35.29**	7.98**	6.84**	3.60**


*n = 264; ^∗^p < 0.05, ^∗∗^p < 0.01*.

**Table 4:** Results of bootstrap for the indirect effect of supervisor feedback environment on creative performance via positive affect (t2) or positive affect change.

Mediator	Effect	*SE*	*z*	*p*	LL 95% CI	UL 95% CI
Positive affect (t2)	0.12	0.05	2.66	0.01	0.04	0.23
Positive affect change	0.06	0.03	2.15	0.03	0.02	0.13


**FIGURE 2:**
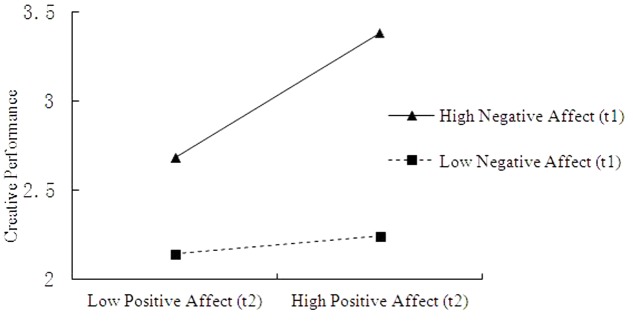
Simple slopes of positive affect (t2) predicting creative performance at low (1 SD below *M*) and high (1 SD above *M*) levels of negative affect (t1).

To test the moderation and moderated mediation hypotheses, we used the procedure developed by Preacher et al. (2007). As shown in **Table 3**, the results indicate that positive affect (t2) and negative affect (t1) have a statistically significant influence (model 2: β = 0.37, *p* < 0.05) on creative performance. According to Preacher et al. (2007), this result implies that the direct effect of positive affect (t2) on creative performance is moderated by negative affect (t1). To fully support Hypothesis 5, this interaction should conform to the hypothesized pattern. Therefore, we applied conventional procedures for plotting simple slopes at one standard deviation above and below the mean of the negative affect (t1) measure (**Figure 2**). Consistent with our expectations, the slope of the relationship between positive affect (t2) and creative performance was relatively stronger for with higher levels of negative affect (t1) than for those with lower levels of negative affect (t1). Results in **Table 5** also show that, the relationship between positive affect (t2) and creative performance was stronger for those with higher levels of negative affect (t1). The Johnson–Neyman technique indicated that for individuals with scores greater than 2.06 on negative affect (t1), positive affect (t2) significantly influenced creative performance. Hypothesis 5 was supported.

**Table 5:** Results for conditional indirect effect of positive affect (t2) on creative performance across levels of negative affect (t1).

Mediator	Level of negative affect (t1)	Mean	Conditional indirect effect	*SE*	LL 95% CI	UL 95% CI
Positive affect (t2)	-1 SD	1.55	0.01	0.03	-0.04	0.08
	*M*	2.14	0.05	0.03	0.01	0.12
	+1 SD	2.74	0.08	0.04	0.02	0.19


**FIGURE 3:**
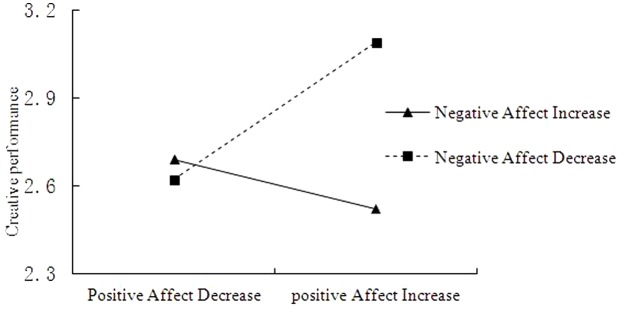
Simple slopes of positive affect increase predicting creative performance at low (1 SD below *M*) and high (1 SD above *M*) levels of negative affect decrease.

As **Table 3** shows, the results indicate a statistically significant interaction between positive affect change and negative affect change (model 4: β = -0.72, *p* < 0.05) that affects creative performance. According to Preacher et al. (2007), this suggests that the direct impact of the positive affect change on creative performance is moderated by negative affect change. To fully support Hypothesis 5, this interaction should conform to the hypothesized pattern. Therefore, we applied conventional procedures for plotting simple slopes at one standard deviation above and below the mean of the negative affect change measure (**Figure 3**). Consistent with our expectations, the slope of the relationship between positive affect increase and creative performance was relatively stronger for negative affect decreases than for negative affect increases. Results in **Table 6** also show that, the relationship between positive affect increase and creative performance was relatively stronger for negative affect decreases. The Johnson–Neyman technique indicated that for individuals with negative affect decreases greater than 0.41, positive affect (t2) significantly influenced creative performance. Hypothesis 9 was supported.

**Table 6:** Results for conditional indirect effect of positive affect change on creative performance across levels of negative affect change.

Mediator	Level of negative affect change	Mean	Conditional indirect effect	*SE*	LL 95% CI	UL 95% CI
Positive	-1 SD	-0.65	0.04	0.02	0.01	0.10
Affect	M	-0.2	0.01	0.02	-0.01	0.06
Change	+1 SD	0.25	-0.02	0.02	-0.08	0.02


## Discussion

In response to the call (George and Zhou, 2007; Bledow et al., 2013), the purpose of this study was to move toward a balanced and dynamic account of the roles of positive and negative affect in the supervisor feedback environment to impact creative performance. Our results indicate that employees’ perceptions of a supportive supervisor feedback environment indirectly influenced their level of creative performance through positive affect (t2). Negative affect (t1) moderated the relationship between positive affect (t2) and creative performance (t2), rendering it more positive if the negative affect (t1) was high. Positive affect change mediated the relationship between the supervisor feedback environment and creative performance; a decrease in negative affect moderated the relationship between an increase in positive affect and creative performance, rendering the relationship more positive if the decrease in negative affect was high.

### Theoretical Contribution

The present study advances several perspectives proposed in previous studies. First, our exploration of the influence of feedback on affect as a multi-dimensional variable helps to resolve the inconsistencies of previous studies that resulted from considering feedback from only a single dimension. Previous studies supported feedback as a one-dimensional influence on various affects, while we considered it as a more comprehensive concept; however, the influence of the feedback environment on positive affect is consistent with previous results (Ilgen et al., 1979; Madjar et al., 2002). This consistency suggests that the supervisor feedback environment should include supportive factors, such as encouraging staff to actively seek feedback, improving the quality of their feedback, and offering feedback with more consideration of employees’ psychological experiences. These types of situational supporting factors can stimulate an individual’s positive affect in ways that promote creative performance (Madjar et al., 2002). The effect on negative affect is not consistent with Ilgen et al. (1979) because Ilgen et al. (1979) focused on only negative feedback and this study focuses on the feedback environment, which includes both negative feedback and other dimensions. In particular, the present study emphasizes the accuracy of negative feedback. These factors contribute to the complexity of the influence mechanism, although our findings are consistent with those of Madjar et al. (2002), who observed that negative affect does not play a mediating role between the supporting environment and creative performance.

Second, we analyzed the affect mechanism between the feedback environment and creative performance from a dynamic affect perspective. On the advice of George and Zhou (2007), this study focuses on the interaction between positive and negative affect. Our results indicate that high creative performance results if negative affect in the morning is followed by a decrease in negative affect and an increase in positive affect throughout the day. Although negative affect can lay the foundation for creativity at a later point in time, at any given moment, the presence of negative affect impedes rather than enables creativity (Baumann and Kuhl, 2002). Negative affect leads to the focusing of cognitive processes on isolated details and promotes a slow and sequential mode of cognitive processing (Derryberry and Tucker, 1994). When negative affect decreases, the focus of cognitive processing expands, which enables the individual to make associations among remotely connected concepts (Baumann and Kuhl, 2002). A dynamic perspective on the feedback environment-creativity link thus suggests that the shift of negative affect plays a core role in achieving high levels of creative performance. On the one hand, people must be capable of tolerating episodes of negative affect; conversely, the ability to down-regulate negative affect is critical (Bledow et al., 2013).

Third, both positive and negative affect play important roles because they are associated with distinct cognitive functions that can contribute to creativity. Positive affect regulates whether cognition proceeds in a controlled, slow, and sequential mode (low positive affect) or in an automatic, fast, and parallel mode (high positive affect). Negative affect regulates whether attention is narrow and focused on isolated elements (high negative affect) or broad and inclusive of the context (low negative affect) (Baumann and Kuhl, 2002). This proposition converges with George and Zhou’s (2007) dual-tuning model and is consistent with Bledow et al. (2011), who observed that software engineers exhibited high levels of work engagement if they experienced a sequence of negative events, such as failures, followed by a positive mood. Negative affect can contribute to creativity because it focuses cognitive processing on discrepant information, thus allowing the individual to develop a detailed and objective understanding of a situation. An affective shift activates associative networks of memory so that new associations can be formed.

### Practical Contribution

The primary findings of this study have important implications for management practices. The main relevant finding of the study is that affect is important. The literature on constraint and on the role demands suggests that positivity alone is not sufficient or the only avenue (Patterson et al., 2011). Positive affect and negative affect play important role in inducing creative performance.

First, positive affect and increasing positive affect are necessary. Shaping a supportive supervisor feedback environment is quite important for improving creative performance and generating positive affect. Leaders should strive to build a supportive feedback environment, improve feedback credibility, encourage support staff to actively seek feedback, improve the quality of the feedback they provide and offer feedback with greater consideration of employees’ psychological experiences. Organizations may develop contexts that support creativity by training employees to provide one another with well-constructed feedback and encouraging employees to seek feedback from one another rather than limiting themselves to supervisor-delivered feedback. As Shalley and Zhou (2008) discussed, such contexts may be developed by setting creativity goals, making creativity a job requirement and building reward systems that value employee creativity.

Second, the context and employee-self must be capable of tolerating negative affect and improving the ability to down-regulate negative affect. From our perspective, a one-sided focus on positive affect and increasing positive affect to improve creative performance is ill-advised. Employees may face different challenges in improving their creative performance depending on how they regulate their affect, and different strategies may be effective. People who remain for a prolonged period in the cognitive processing mode that is induced by negative affect may benefit from strategies that facilitate the down-regulation of negative thoughts and feelings, such as self-relaxation and seeking a socially supportive work environment (e.g., Grossman et al., 2004). In contrast, the creative performance of people who quickly down-regulate negative affect may benefit from an increased tolerance of negative thoughts and feelings so that negative affect is not brushed aside too quickly. A deliberate focus on information that elicits negative affect may be effective for encouraging such individuals to question their preferred alternatives or to reflect on barriers that could hinder their pursuit of goals (Oettingen et al., 2005).

### Limitations and Future Research Suggestions

Although our findings have contributed answers to several recent questions in the feedback-creative performance literature, this study has several limitations.

One limitation is that the small sample size reported here may have affected the current results. However, this small sample size coupled with the significant results suggests that the current findings are reliable. Our data were from an organization in China; therefore, the external validity of our findings may not be accurate in other countries. Our analysis does not preclude various interpretations in other settings because organizational or cultural differences may influence the attitudes and behaviors of employees. Future studies should utilize larger samples, which will render the results more specific and representative. To improve the generalizability of our results, future research should apply our model to multi-organizational and cross-national samples.

The second limitation lies in the data collection method. Regarding creative performance, we argue that leader reports may be the most valid means of measuring a person’s creativity on a particular work day (Shalley et al., 2009). Thus, these results could be biased by common method variance (CMV) even though procedural and statistical efforts were made to address these concerns, thus alleviating some of this threat. In the future, for dealing with CMV, research should identify new means for objectively measuring creative performance or should consider what biases may be present. Some procedural remedies in designing the scale and using different scale types can reduce the likelihood of CMV. Some statistical remedies to detect and control for CMV, like a *post hoc* Harman one-factor analysis. Regarding feedback environment, although the longitudinal method is used for demonstrating the causal relationship between variables, feedback environment is not tied to any particular time or day, but the affect was measured on 1 day at two different times. Future research should use stimulated intervention method to change different latent variables of feedback environment, and find the different level of affect and creative performance.

Third, we argued that negative affect may contribute to creativity because it focuses cognitive processing on discrepant information, allowing a person to develop a detailed and objective understanding of a situation. We further proposed that an affective shift activates associative networks of memory and leads to the formation of new associations. However, because we did not measure the participants’ cognitive processes or the creative output, the relative contribution of these mediating processes and their interplay could not be examined. Further research can examine how the overall influence of affect change on cognitive functioning affects the processing of specific cognitive content, such as the identification and elaboration of work-related problems and the generation of creative solutions. Unconscious processes that take place during a subsequent incubation phase, in which attention is focused away from the problem, can influence creativity (Dijksterhuis and Meurs, 2006).

Fourth, the development of new and useful ideas within the time frame of a single day should not imply that people discuss or implement ideas immediately. Creative performance on any particular day is not always observed by others or reflected in objective outcomes. A further research question concerns the time frames in which an affective shift occurs and whether a shift has similar consequences across different time frames. Potential time frames can last from milliseconds to years, and affective shifts that occur in different time frames are interwoven. An artist, for instance, may reach a period of peak creativity after emerging from a long-lasting crisis (Jamison and Scogin, 1995). During the period of peak creativity, there may be short-term affective shifts that influence creative performance on specific pieces of art.

## Author Contributions

ZG provided substantial contributions to the research conception and design. ZG analyzed and interpreted the data. ZG and NZ wrote the paper, NZ provided critical revisions of the paper. ZG and NZ both attended to the revision of the paper. ZG and NZ both approved of this version of the paper to be published.

## Conflict of Interest Statement

The authors declare that the research was conducted in the absence of any commercial or financial relationships that could be construed as a potential conflict of interest.
